# Muscle wasting and adipose tissue browning in infantile nephropathic cystinosis

**DOI:** 10.1002/jcsm.12056

**Published:** 2015-09-07

**Authors:** Wai W. Cheung, Stephanie Cherqui, Wei Ding, Mary Esparza, Ping Zhou, Jianhua Shao, Richard L. Lieber, Robert H. Mak

**Affiliations:** ^1^ Department of PediatricsUniversity of CaliforniaSan DiegoCAUSA; ^2^ Division of Nephrology, The 5th People's Hospital of ShanghaiFudan UniversityShanghaiChina; ^3^ Department of Orthopedic SurgeryUniversity of CaliforniaSan DiegoCAUSA; ^4^ Department of PediatricsThe 2nd Hospital of Harbin Medical UniversityHarbinChina; ^5^ Rehabilitation Institute of ChicagoChicago

**Keywords:** Infantile nephropathic cystinosis, Adipocyte browning, Energy homeostasis, Muscle wasting, Cachexia

## Abstract

**Background:**

Muscle wasting is a common complication in patients with infantile nephropathic cystinosis, but its mechanism and association with energy metabolism is not known. We define the metabolic phenotype in Ctns^−/−^ mice, an established murine model of infantile nephropathic cystinosis, with focus on muscle wasting and energy homeostasis.

**Methods:**

Male Ctns^−/−^ mice and wild‐type (WT) controls were studied at 1, 4, 9, and 12 months of age. As Ctns^−/−^ mice started to develop chronic kidney disease (CKD) at 9 months of age, 9‐ and 12‐month‐old Ctns^−/−^ mice were also compared with age‐matched WT mice with CKD. Serum and urine chemistry and energy homeostasis parameters were measured. Skeletal muscle histomorphometry and *in vivo* muscle function were measured. We studied expression of genes involved in muscle mass regulation, thermogenesis, energy metabolism, adipogenesis, and adipose tissue browning in Ctns^−/−^ mice.

**Results:**

Ctns^−/−^ mice showed loss of weight and lean mass and increased energy expenditure. Ctns^−/−^ mice exhibited abnormal energy homeostasis before the onset of their CKD. Food intake in Ctns^−/−^ mice was comparable with age‐matched WT controls. However, significantly lower total body mass starting at 1 month of age and increased energy expenditure at 4 months of age preceded the onset of CKD at 9 months of age in Ctns^−/−^ mice. Muscle accept content in 1‐ and 4‐month‐old Ctns^−/−^ mice was significantly lower than that in age‐matched WT controls. At 12 months of age, muscle fibre area and *in vivo* muscle strength was reduced in Ctns^−/−^ mice than that in WT or CKD controls. Muscle wasting in Ctns^−/−^ mice was associated with inhibition of myogenesis, activation of muscle proteolysis pathways, and overexpression of pro‐inflammatory cytokines. Increased energy expenditure was associated with elevation of thermogenesis in skeletal muscle and adipose tissues. The development of beige adipocytes in Ctns^−/−^ mice is a novel finding. Expression of beige adipose cell surface markers (CD137, Tmem26, and Tbx1) and uncoupling protein‐1, which is a brown adipose tissue marker, was observed in inguinal white adipose tissue of Ctns^−/−^ mice. Expression of key molecules implicated in the pathogenesis of adipose tissue browning (Cox2, cytochrome c oxidase subunit II; PGF2α, prostaglandin F2α; IL‐1α, interleukin 1α; IL‐6, interleukin 6; TNF‐α, tumor necrosis factor α) was significantly increased in inguinal white adipose tissue of Ctns^−/−^ mice than that in WT controls.

**Conclusion:**

This study describes a mouse model of nephropathic cystinosis presenting with profound muscle wasting. The mechanism for hypermetabolism in Ctns^−/−^ mice may involve up‐regulation of thermogenesis pathways in skeletal muscle and adipose tissues. This study demonstrates, for the first time, the development of beige adipocytes in Ctns^−/−^ mice. Understanding the underlying mechanisms of adipose tissue browning in cystinosis may lead to novel therapy.

## Introduction

Cystinosis is a rare autosomal recessive disorder caused by mutations of the *CTNS* gene (17p13) encoding the lysosomal cystine transporter, cystinosin.^1^ This results in the intralysosomal accumulation of cystine in all tissues, most notably the kidneys. Patients with infantile nephropathic cystinosis (INC) exhibit signs and symptoms of renal Fanconi syndrome and chronic kidney disease (CKD) in early childhood.^2^ Muscle wasting is a common complication in patients with cystinosis. The prevalence of muscle weakness and myopathy varies from 33 to 60% in long‐term follow‐up studies of patients with INC.^3^, ^4^, ^5^ These complications negatively affect the quality of life due to decreased mobility and are associated with terminal events such as swallowing difficulty and respiratory musculature weakness leading to aspiration pneumonia, respiratory failure, and death.^2^, ^6^ The underlying mechanism of muscle wasting in patients with INC is not well understood.^2^, ^7^, ^8^ In this study, we characterize the metabolic phenotype in Ctns^−/−^ mice, an established murine model of INC, with focus on muscle wasting and energy homeostasis.

## Materials and methods

### Mice

C57BL/6 Ctns^−/−^ mice were provided by Professor Corinne Antignac. Wild‐type (WT) C57BL/6 control mice were acquired from Jackson Lab. Only male mice were used for this study. CKD was surgically induced in C57BL/6 mice by five‐sixth nephrectomy.^9^ The study protocol was in compliance with Institutional Animal Care and Use Committee and National Institute of Health guidelines for the care and use of laboratory animals.

### Serum and urine chemistry

Urine and serum phosphate, blood urea nitrogen (BUN), and bicarbonate levels were measured by standard laboratory methods (*Ta* *ble* 1). Urine and serum creatinine levels were analysed using QuantiChrom creatinine assay kit (BioAssay Systems). Serum cystatin C levels were measured by enzyme‐linked immunosorbent assay method (part number: ALX‐850‐328, Enzo Life Sciences). Protein levels in urine were measured using Pierce BCA protein assay kit. Tubular excretion of phosphorus (TEP) index was calculated according to the formula: [Phosphorus_urine_ ÷ Creatinine_serum_] × [Phosphorus_serum_ ÷ Creatinine_urine_] × 100.

**Table 1:** Urine and serum chemistry in mice during the course of the 12‐month study

	WT control	Ctns^−/−^	WT control	Ctns^−/−^	WT control	Ctns^−/−^	CKD control	WT control	Ctns^−/−^	CKD control
	1 month	1 month	4 months	4 months	9 months	9 months	9 months	12 months	12 months	12 months
Urine chemistry										
Phosphate (µmol/24 h)	2.4 ± 0.5	3.5 ± 0.4	2.5 ± 0.3	4.1 ± 0.5^b^	3.7 ± 0.5	4.7 ± 0.2^b^	4.5 ± 0.2^b^	3.9 ± 0.2	6.5 ± 0.4^b^	5.9 ± 0.3^b^
TEP (%)	12.2 ± 2.5	12.1 ± 2.1	10.6 ± 2.6	22.1 ± 5.4^b^	17.8 ± 3.2	52.8 ± 6.7^b^	43.7 ± 2.1^b^, ^c^	16.4 ± 4.3	78.9 ± 12.3^b^	53.4 ± 2.3^b^, ^c^
Protein (mg/24 h)	6.7 ± 0.6	5.7 ± 0.3	8.4 ± 0.3	7.8 ± 0.5	10.8 ± 1.1	13.2 ± 0.6^b^	12.5 ± 0.4^b^	13.8 ± 0.8	17.4 ± 1.1^b^	15.7 ± 0.2^b^
Volume (mL/24 h)	0.4 ± 0.1	0.5 ± 0.2	0.6 ± 0.3	0.8 ± 0.4	0.8 ± 0.2	0.9 ± 0.3	0.8 ± 0.1	0.6 ± 0.2	1.6 ± 0.2^b^	0.7 ± 0.3
Serum chemistry										
BUN (mg/dL)	32.9 ± 2.1	32.8 ± 1.4	32.7 ± 4.4	36.5 ± 2.2	35.6 ± 3.4	54.8 ± 3.4^b^	45.7 ± 2.4^b^	31.7 ± 3.5	65.9 ± 4.7^b^	51.5 ± 2.3^b^, ^c^
Creatinine (mg/dL)	0.28 ± 0.04	0.23 ± 0.06	0.29 ± 0.07	0.29 ± 0.06	0.34 ± 0.05	0.43 ± 0.09	0.36 ± 0.11	0.31 ± 0.03	0.52 ± 0.05^b^	0.42 ± 0.06^b^
Creatinine clearance (μL/min)	65.4 ± 5.6	64.2 ± 9.5	58.9 ± 7.4	55.9 ± 6.3	67.8 ± 7.3	55.3 ± 6.7	59.8 ± 2.5	72.3 ± 4.7	32.6 ± 3.6^a^	52.8 ± 2.6^a^, ^c^
Cystatin C (mg/dL)	0.08 ± 0.02	0.08 ± 0.02	0.09 ± 0.03	0.07 ± 0.03	0.19 ± 0.04	0.36 ± 0.05^b^	0.31 ± 0.03^b^	0.11 ± 0.03	0.46 ± 0.11^b^	0.34 ± 0.03^b^
Bicarbonate (mmol/L)	26.8 ± 1.6	26.1 ± 0.9	27.9 ± 2.2	26.6 ± 1.5	28.5 ± 2.1	25.9 ± 1.6	26.7 ± 1.1	26.5 ± 2.4	26.3 ± 1.8	27.3 ± 1.7

Ctns^−/−^ mice were compared with age‐matched wild‐type (WT) controls. In addition, 9‐ and 12‐month‐old Ctns^−/−^ mice were also compared with age‐matched CKD mice. Number of mice is ≥8 at each time point. Data are expressed as mean ± SEM. BUN, blood urea nitrogen; TEP, tubular excretion of phosphorus.

a *P* < 0.05, significantly lower in Ctns^−/−^ or CKD mice vs. WT controls.

b *P* < 0.05, significantly higher in Ctns^−/−^ or CKD mice vs. WT controls.

c *P* < 0.05, significantly different between Ctns^−/−^ or CKD mice.

### Indirect calorimetry

Indirect calorimetry was performed in mice using Oxymax calorimetry (Columbus Instruments).^9^ Oxygen (VO_2_) and carbon dioxide (VCO_2_) consumption were measured. The respiratory exchange ratio (RER) was calculated as the quotient VCO_2_/VO_2_. Energy expenditure was measured as a production of kilocalorie of heat and was calculated as Caloric Value (CV) × VO_2_, where CV is 3.815 + 1.232 × RER.^10^

### Measurement of body composition

Whole body fat mass and lean mass of mice were determined by quantitative magnetic resonance analysis (EchoMRI‐100™, Echo Medical Systems).^11^

### Muscle fibre size

Excised soleus and tibialis anterior muscles were snap‐frozen in isopentane cooled by liquid nitrogen and stored at −80°C for subsequent analysis. Muscle cross sections (10 µm thick) were taken from muscle midbelly. Sections were first treated with 1% bovine serum albumin and normal goat and mouse serum as blocking agents. Sections were incubated overnight with a polyclonal anti‐laminin antibody (Sigma, dilution 1:1000) and then with the secondary antibody, Alexa Fluor 594 goat anti‐rabbit immunoglobulin G (Invitrogen, dilution 1:200). The laminin antibody was used to label the fibre perimeter and facilitate fibre area quantification. Sections were imaged with a microscope (Leica CTR 6500, Buffalo Grove) fit with a fluorescent camera (Leica DFC365 FX) set for 594 emission fluorescence using a 10× objective. Fibre cross‐sectional areas were measured using a custom‐written macro in ImageJ (NIH). Filtering criteria were applied to ensure measurement of actual muscle fibres.^12^ These criteria rejected regions with areas below 50 µm^2^ and above 5000 µm^2^ to eliminate neurovascular structures and ‘optically fused’ fibres, respectively.

### Muscle grip strength and rotarod activity

Grip Strength Meter (model 47106, UGO Basile) and AccuRotor Rota Rod (model RRF/SP, Accuscan Instrument) were used to assess forelimb grip strength and motor coordination in mice, respectively.^13^

### Uncoupling proteins and pro‐inflammatory cytokines

Uncoupling protein (UCP) contents in tissue were assayed using mouse UCP‐1 (E95557Mu, Uscn Life Science), UCP‐2 (E2066m, EIAab), and UCP‐3 (E2068m, EIAab) assay kits, respectively. Sample protein concentration was determined by Pierce BCA protein assay kit. Muscle tissue lysate protein levels of IL‐1α, IL‐1**β**, IL‐6, and TNF‐**α** were quantified with Mouse Quantibody Custom Array (RayBiotech).

### Tissue adenosine triphosphate content

Adenosine triphosphate (ATP) concentrations in tissue homogenates were assayed using the ATP colorimetric/fluorometirc assay kit (ab83355, Abcam).

### Quantitative real‐time PCR

RNA was isolated from extracted tissue by using TriZol (Life Technology) and further purified with Direct‐zol RNA MiniPre Kit (Zymo Research). cDNA was synthesized using SuperScript III Reverse Transcriptase and oligo(dT)_12‐18_ primer (Invitrogen). Transcriptional levels of target genes were measured by real‐time PCR, using a 7300 Real‐Time PCR System (ABI Applied Biosystems). Appropriate primers and probes for target genes were listed (*Tables* 2 and 3). Comparative 2^−ΔΔCt^ method was used to determine the relative quantification of target gene. Final results were expressed in arbitrary units, with one unit being the mean mRNA level in the age‐matched WT control mice.

**Table 2:** PCR primer information

Gene	Forward primer sequence	Reverse primer sequence	Primer bank ID
Acox1	TAACTTCCTCACTCGAAGCCA	AGTTCCATGACCCATCTCTGTC	26333821a1
Acsl1	TGCCAGAGCTGATTGACATTC	GGCATACCAGAAGGTGGTGAG	31560705a1
Atg1	AGATGAAAGCAAGATGTTGCCT	CCCTGTAGGTCAGCCATATTCTA	27923915a1
Atrogin‐1	CAGCTTCGTGAGCGACCTC	GGCAGTCGAGAAGTCCAGTC	13385848a1
CD137	CGTGCAGAACTCCTGTGATAAC	GTCCACCTATGCTGGAGAAGG	20306992a1
Cidea	TGACATTCATGGGATTGCAGAC	GGCCAGTTGTGATGACTAAGAC	6680944a1
CPT1α	CTCCGCCTGAGCCATGAAG	CACCAGTGATGATGCCATTCT	27804309a1
CPT1β	GCACACCAGGCAGTAGCTTT	CAGGAGTTGATTCCAGACAGGTA	6753512a1
Dio2	AATTATGCCTCGGAGAAGACCG	GGCAGTTGCCTAGTGAAAGGT	6753638a1
Gapdh	AGGTCGGTGTGAACGGATTTG	TGTAGACCATGTAGTTGAGGTCA	6679937a1
Glut1	GCCTGACCTTCGGATATGAGC	TGCCATAGCAGTCAATGAGGA	18485498a1
Glut4	GTGACTGGAACACTGGTCCTA	CCAGCCACGTTGCATTGTAG	6678015a1
Hsl	CCAGCCTGAGGGCTTACTG	CTCCATTGACTGTGACATCTCG	26325924a1
IGF‐I	CTGGACCAGAGACCCTTTGC	GGACGGGGACTTCTGAGTCTT	6754308a1
MyoD	CCACTCCGGGACATAGACTTG	AAAAGCGCAGGTCTGGTGAG	6996932a1
Myogenin	GAGACATCCCCCTATTTCTACCA	GCTCAGTCCGCTCATAGCC	13654247a1
Myostatin	AGTGGATCTAAATGAGGGCAGT	GTTTCCAGGCGCAGCTTAC	6754752a1
MuRF‐1	GTGTGAGGTGCCTACTTGCTC	GCTCAGTCTTCTGTCCTTGGA	21523717a1
Pax‐3	CCGGGGCAGAATTACCCAC	GCCGTTGATAAATACTCCTCCG	26377023a1
Pax‐7	TCTCCAAGATTCTGTGCCGAT	CGGGGTTCTCTCTCTTATACTCC	34328055a1
Pgc1α	TATGGAGTGACATAGAGTGTGCT	CAGGAGTTGATTCCAGACAGGTA	6679433a1
Pgc1β	TCCTGTAAAAGCCCGGAGTAT	GCTCTGGTAGGGGCAGTGA	18875426a1
Pparα	AGAGCCCCATCTGTCCTCTC	ACTGGTAGTCTGCAAAACCAAA	31543500a1
Pparδ	CAAGTGGGGTCAGTCATGGAA	GCTGGAAGGAAGCGTGTGTT	403943a1
Prdm16	CCCCACATTCCGCTGTGAT	CTCGCAATCCTTGCACTCA	124107622c3
Tbx1	CTGTGGGACGAGTTCAATCAG	TTGTCATCTACGGGCACAAAG	22094109a1
Tmem26	TTCCTGTTGCATTCCCTGGTC	GCCGGAGAAAGCCATTTGT	29244332a1

Appropriate primer sequence was obtained from http://pga.mgh.harvard.edu/primerbank/

**Table 3:** Taqman gene expression assays‐on‐demand identities

Target genes	Assay identities
Cox2	Mm03294838_g1
IL‐1α	Mm00494938_m1
IL‐6	Mm00446190_m1
PGF2α synthase	Mm00442792_m1
TNF‐α	Mm00443258_m1

Internal control gene	Assay Identities
Gapdh	4352339E

### Statistical analysis

Results are reported as mean ± standard error of the mean (SEM). The means of variables were compared with Student's *t*‐test, assuming unequal variances, or Welch's analysis of variance, when more than two groups were compared. In that case, a pair‐wise *t*‐test with Bonferroni correction was performed. The Wilcoxon rank‐sum test was used to compare the median of variables. All tests were two‐sided. A *P* value of <0.05 was considered significant. Analyses were performed using SPSS 16.0 for Macintosh.

## Results

### Urine and serum chemistry in Ctns^−/−^ mice

We characterized urine and serum chemistry in Ctns^−/−^ mice compared with age‐matched WT mice at the age of 1, 4, 9, and 12 months. In Ctns^−/−^ mice, significantly higher urine phosphate levels and TEP were demonstrated as early as 4 months old relative to controls (*Table* 1). Proteinuria was evident at 9 months old while polyuria was significant at 12 months old Ctns^−/−^ mice. BUN and serum cystatin C levels were significantly higher in Ctns^−/−^ mice than controls at 9 months of age. Serum creatinine was significantly higher while creatinine clearance was decreased in Ctns^−/−^ mice relative to WT controls at 12 months of age. Serum bicarbonate levels were not different between Ctns^−/−^ mice and WT controls.

As Ctns^−/−^ mice developed CKD at the age of 9 months, urine and serum chemistry in 9‐ and 12‐month‐old Ctns^−/−^ mice were compared with age‐matched pair‐fed CKD mice. TEP, volume of 24 h urine excretion, and BUN were elevated while serum creatinine clearance was decreased in 9‐ and 12‐month‐old Ctns^−/−^ mice than that in CKD mice (*Table* 1).

### Lower body weight, increased energy homeostasis, and muscle wasting in Ctns^−/−^ mice

**Figure 1:**
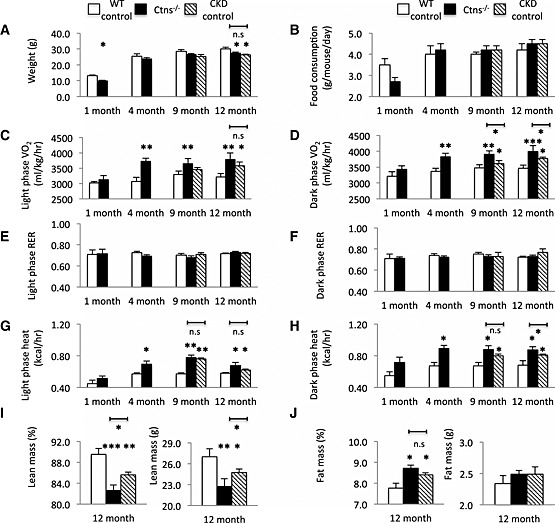
Energy homeostasis and body composition in Ctns−/− mice. Body mass (*A*) and food consumption (*B*); resting metabolic rate (VO_2_) of light and dark phase (*C* and *D*); light and dark phase respiratory exchange ratio (RER) (*E* and *F*); and light and dark phase energy expenditure (*G* and *H*). Mice were scanned by quantitative magnetic resonance (QMR) at 12 months of age. Percentage of lean mass, total lean mass, percentage of fat mass, and total fat mass (*I* and *J*). Number of mice is ≥8 in each group. Data are expressed as mean ± standard error of the mean. *P < 0.05, **P < 0.01, ***P < 0.001.

Body mass of Ctns^−/−^ mice at 1 month old was 25.8% lighter than age‐matched WT controls. At 12 months old, Ctns^−/−^ mice were 9.0% lighter than WT controls (*Figure* 1A). Decreased body weight in Ctns^−/−^ mice did not appear to result from reduced food intake. All mice were fed *ad libitum*, and food consumption in Ctns^−/−^ mice was not different from WT controls at 1, 4, 9, and 12 months of age (*Figure* 1B). To further investigate the impact of cystinosis on muscle wasting and energy homeostasis beyond the detrimental effects of renal dysfunction, age‐matched CKD mice were pair‐fed to 9‐ and 12‐month‐old Ctns^−/−^ mice (*Figure* 1B). Body mass of 9‐ and 12‐month‐old Ctns^−/−^ mice was comparable with that of pair‐fed CKD mice (*Figure* 1A). Ctns^−/−^ mice demonstrated hypermetabolism—their rate of oxygen consumption (VO_2_) and heat production in both light and dark phases was significantly higher than that of WT controls as young as 4 months old (*Figure* 1C, 1D, 1G, and 1H). VO_2_ consumption and heat production in dark phase was significantly elevated in 9‐ and 12‐month‐old Ctns^−/−^ mice than that in pair‐fed CKD mice, respectively (*Figure* 1D and 1H). There was no difference in RER between Ctns^−/−^, WT, and CKD mice (*Figure* 1E and 1F). At the end of the study, we used quantitative magnetic resonance to analyze body composition. Muscle wasting was evident in Ctns^−/−^ and CKD mice vs. WT mice. At 12 months of age, the percentage of lean mass and total lean mass was significantly decreased in Ctns^−/−^ than WT or CKD mice (*Figure* 1I). In contrast, the percentage of fat mass was elevated in Ctns^−/−^ mice vs. WT mice while the total fat mass was not different between Ctns^−/−^, WT, and CKD mice (*Figure* 1J).

### Reduced muscle fibre size and impaired muscle function in Ctns^−/−^ mice

**Figure 2:**
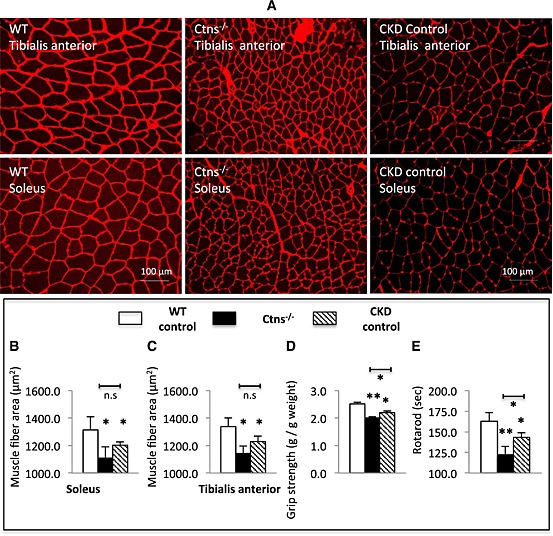
Skeletal muscle histomorphometry and muscle function in 12‐month‐old Ctns−/− mice. Representative photomicrographs of soleus and tibialis anterior immunohistochemical sections labeled with a polyclonal antibody to laminin with magnification ×200 (*A*). Soleus muscle and tibialis anterior muscle average fiber area in each group (*B*) and (*C*), respectively. Forelimb grip strength (*D*) and motor coordination (*E*). Number of mice is ≥8 in each group. Data are expressed as mean ± standard error of the mean. *P < 0.05, **P < 0.01.

We studied the effects of cystinosis on skeletal muscle histomorphometry. Muscle sections were labeled for the muscle fibre basement membrane. Representative photomicrographs of muscle sections in 12‐month‐old Ctns^−/−^ mice and WT and CKD controls are shown in *Figure* 2A. Mean soleus and tibias anterior fibre cross‐sectional area in Ctns^−/−^ mice was 84.5 and 85.3% of that observed in WT controls (*Figure* 2B and 2C). Muscle wasting in Ctns^−/−^ mice was associated with progressive skeletal muscle weakness. Muscle function, as assessed by forelimb grip strength and rotarod activity, was significantly decreased in Ctns^−/−^ mice vs. WT or CKD controls (*Figure* 2D and 2E).

### Muscle wasting signaling in Ctns^−/−^ mice

**Figure 3:**
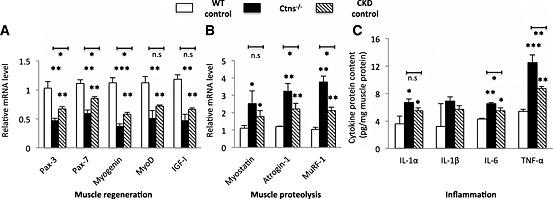
Gene expression of key molecules implicated in muscle wasting in 12‐month‐old Ctns−/− mice (*A* and *B*) and muscle pro‐inflammatory cytokine protein contents (*C*). Comparative 2‐ΔΔCt method was used to determine the relative quantification of genes in muscle. To normalize each sample for RNA content, the internal control gene GAPGH was used. Final results were expressed in arbitrary units, with one unit being the mean mRNA level in the wild‐type controls. Data are expressed as mean ± standard error of the mean. *P < 0.05, **P < 0.01, ***P < 0.001.

We investigated the signaling pathways associated with skeletal muscle wasting in 12‐month‐old Ctns^−/−^ mice. Gastrocnemius muscle from experimental mice was dissected, and total RNA from gastrocnemius muscles was extracted and reversely transcribed. Gene expression for several transcripts associated with myogenesis and skeletal regeneration (Pax‐3, Pax‐7, Myogenin, MyoD, and IGF‐I) was significantly decreased in gastrocnemius muscle of Ctns^−/−^ mice relative to WT controls (*Figure* 3A). Gene expression of Pax‐3, Pax‐7, and Myogenin in Ctns^−/−^ mice was significantly lower than that in CKD mice. In contrast, expression of muscle proteolytic genes, Myostatin, Atrogin‐1, and MuRF‐1, was significantly increased in Ctns^−/−^ mice than in WT controls (*Figure* 3B). In addition, muscle Atrogin‐1 and MuRF‐1 gene expression in Ctns^−/−^ mice was higher than that in CKD controls. Muscle lysate protein levels of inflammatory cytokines (IL‐1α, IL‐6, and TNF‐α) were increased in Ctns^−/−^ mice than in WT controls (*Figure* 3C). Muscle protein content of IL‐6 and TNF‐α was significantly elevated in Ctns^−/−^ mice than in CKD controls.

### Increased thermogenesis and decreased adenosine triphosphate content in skeletal muscle and adipose tissues of Ctns^−/−^ mice

**Figure 4:**
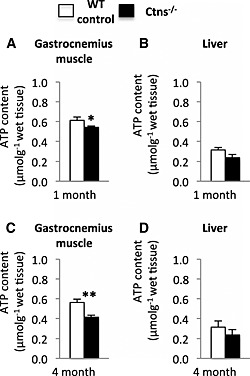
Adenosine triphosphate content in muscle and liver in 1‐ and 4‐month‐old Ctns−/− mice. Adenosine triphosphate content in tissue lysate was measured in duplicate and calculated per gram of tissue. Number of mice is ≥6 in each group. Data are expressed as mean ± standard error of the mean. *P < 0.05, **P < 0.01, ***P < 0.001.

Food consumption of 1‐ and 4‐month‐old Ctns^−/−^ mice was not different than that of age‐matched WT controls (*Figure* 1B). The observed lower body mass in young Ctns^−/−^ mice may be associated with disturbances in energy homeostasis before the onset of renal dysfunction. We measured muscle and liver ATP content in 1‐ and 4‐month‐old Ctns^−/−^ mice vs. WT controls. Gastrocnemius muscle ATP was significantly decreased in Ctns^−/−^ mice relative to age‐matched WT controls (*Figure* 4A and 4C).

**Figure 5:**
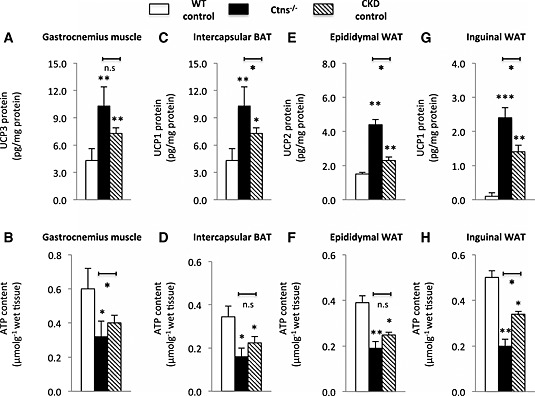
Uncoupling protein level and adenosine triphosphate content in muscle and adipose tissue of 12‐month‐old Ctns−/− mice. Uncoupling protein content (*A*, *C*, *E*, and *G*) and adenosine triphosphate content (*B*, *D*, *F*, and *H*) in tissue were measured. Number of mice is ≥6 in each group. Data are expressed as mean ± standard error of the mean. *P < 0.05, **P < 0.01.

Protein contents of UCPs in gastrocnemius muscle and adipose tissues were elevated in 12‐month‐old Ctns^−/−^ mice relative to WT (*Figure* 5A, 5C, 5E, and 5G). Protein contents of UCP‐1 and UCP‐2 in adipose tissue were increased in 12‐month‐old Ctns^−/−^ mice vs. age‐matched CKD mice. In contrast, ATP contents in muscle and inguinal white adipose tissue (WAT) were markedly decreased in Ctns^−/−^ mice relative to WT and CKD controls (*Figure* 5B and 5H). ATP contents in intercapsular brown adipose tissue (BAT) and epididymal WAT were decreased in Ctns^−/−^ mice than in WT control but were not different than those in age‐matched CKD controls (*Figure* 5D and 5F).

**Figure 6:**
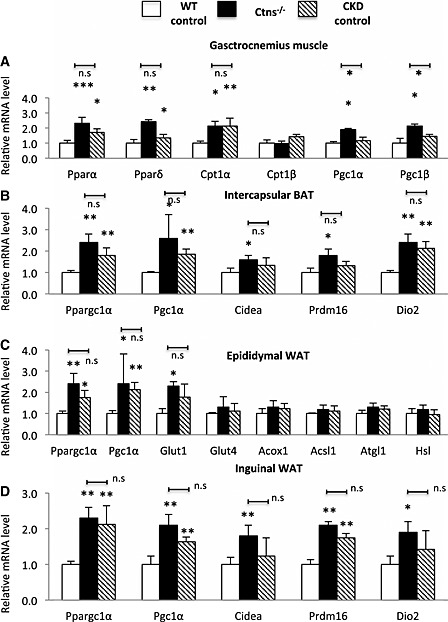
Thermogenic gene expression in 12‐month‐old Ctns−/− mice. Thermogenic gene expression in skeletal muscle and adipose tissues (*A* to *D*). Number of mice = 6. Data are expressed as mean ± standard error of the mean. *P < 0.05, **P < 0.01, ***P < 0.001.

We compared expression profile of genes related to energy consumption in 12‐month‐old Ctns^−/−^ mice. The expression levels of genes related to fatty acid oxidation (Pparα, Pparδ, and Cpt1α) and energy consumption (Pgc1α and Pgc1β) were increased in the skeletal muscle of Ctns^−/−^ mice than in that of WT controls (*Figure* 6A). In addition, Pgc1α and Pgc1β gene expression in Ctns^−/−^ mice was significantly higher than that in CKD mice. Increased thermogenic gene expression (Ppargc1α, Pgc1α, Cidea, Prdm16, and Dio2) was found in BAT of Ctns^−/−^ mice than in WT controls (*Figure* 6B). Epididymal WAT exhibited higher expression of Ppargc1α, Pgc1α, and glucose transporter Glut1 in Ctns^−/−^ mice than in WT controls, but no difference was found in expression of genes involved in lipolytic metabolism (Acox1, Acstl1, Atg1, and Hsl) in Ctns^−/−^ mice relative to WT or CKD mice (*Figure* 6C). Moreover, inguinal WAT of Ctns^−/−^ mice displaced higher expression of Ppargc1α, Pgc1α, Cidea, Prdm16, and Dio2 than that of WT controls (*Figure* 6D).

### Adipose tissue browning in Ctns^−/−^ mice

**Figure 7:**
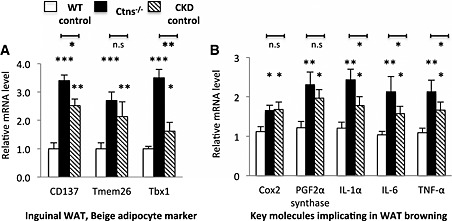
Adipose tissue browning in 12‐month‐old Ctns−/− mice. Gene expression of beige adipocyte marker (CD137, Tmem26, and Tbx1) in inguinal white adipose tissue was measured (*A*). Gene expression of key molecules implicated in adipose tissue browning (*B*). Results were analysed and expressed as in *Figure* 3. *P < 0.05, **P < 0.01, ***P < 0.001.

Beige adipocytes are a distinct type of thermogenic fat cells in mice and humans. Browning of beige adipocytes in WAT has been associated with increased energy expenditure in cachexia. We observed elevated expression of beige adipose cell surface markers (CD137, Tmem26, and Tbx1) in inguinal WAT in 12‐month‐old Ctns^−/−^ mice than in WT controls (*Figure* 7A). Furthermore, inguinal WAT CD137 and Tbx1 expression was higher in Ctns^−/−^ mice than in CKD controls. Another important marker for beige adipocyte in inguinal WAT is UCP‐1, which is usually not detected in WAT. UCP‐1 protein was detected in inguinal WAT of 12‐month‐old Ctns^−/−^ and CKD mice but was undetectable in WT controls (*Figure* 5G). UCP‐1 protein level in inguinal WAT of 12‐month‐old Ctns^−/−^ was higher than that in CKD mice. Collectively, these results demonstrate the development of beige adipocytes in 12‐month‐old Ctns^−/−^ mice. Our results show that increased energy expenditure is associated with adipose tissue browning in Ctns^−/−^ mice.

We also measured gene expression of key molecules implicated in the pathogenesis of WAT browning in mice. Inguinal WAT of 12‐month‐old Ctns^−/−^ mice displayed higher mRNA expression of Cox2 and PGF2α than that of WT controls (*Figure* 7B). Gene expression of inflammatory cytokines, IL‐1α, IL‐6, and TNF‐α, was significantly elevated in inguinal WAT of 12‐month‐old Ctns^−/−^ mice than that in age‐matched WT controls or CKD mice (*Figure* 7B).

## Discussion

This study confirms the presence of the tubular and glomerular dysfunction in Ctns^−/−^ mice. Fanconi syndrome, the hallmark of the tubular dysfunction in children with INC, characterized by phosphaturia, proteinuria, and polyuria, was demonstrated in Ctns^−/−^ mice (*Table* 1). Indeed, elevation of TEP index, the more sensitive parameter of tubular function, was demonstrated earlier in the course of the renal disease (4 months of age) than the glomerular dysfunction (9 months of age). Serum cystatin C is a reliable marker for renal function in mice and humans, which has the advantage over serum creatinine when muscle wasting can be a confounding factor.^14^, ^15^ Serum cystatin C levels were elevated in Ctns^−/−^ mice than in controls at 9 months of age. The time course of renal dysfunction in Ctns^−/−^ mice of this study is in concordance with a recent report.^16^

Importantly, we show that Ctns^−/−^ mice exhibit disturbances in energy homeostasis prior to the development of CKD. Basal metabolic rate comprises 50–80% of daily energy expenditure and exhibits circadian rhythms. Food consumption in Ctns^−/−^ mice was not different than age‐matched WT controls (*Figure* 1B). However, significantly lower total body mass starting at 1 month of age as well as increased energy expenditure at 4 months of age (*Figure* 1A, 1C, 1D, 1G, and 1H) preceded the onset of CKD at 9 months of age in Ctns^−/−^ mice (*Table* 1). Muscle ATP content in 1‐ and 4‐month‐old Ctns^−/−^ mice was significantly lower than that in age‐matched WT controls (*Figure* 4A and 4C). We compared parameters of energy homeostasis in 9‐ and 12‐month‐old Ctns^−/−^ mice vs. pair‐fed age‐matched CKD mice. VO_2_ and energy expenditure at dark phase was significantly elevated in 9‐ and 12‐month‐old Ctns^−/−^ mice than that in CKD controls (*Figure* 1D and 1H).

Muscle wasting is a life‐threatening complication in cystinosis.^2^, ^6^ We observed decreased body mass and lean mass in 12‐month‐old Ctns^−/−^ mice than in WT control or pair‐fed CKD mice (*Figure* 1A and 1I). We further measured muscle fibre histomorphometry and muscle function in Ctns^−/−^ mice. Soleus and tibias anterior muscle were chosen as they represent extremes of mouse muscle types in terms of fibre type composition^17^ since the soleus is composed of about 50% type 1 fibre (slow) while the tibias anterior has no slow fibres. Cross‐sectional area of soleus and tibialis anterior was significantly reduced in Ctns^−/−^ mice than in WT controls (*Figure* 2A, 2B, and 2C). We also showed that reduced muscle mass and decreased muscle fibre cross‐sectional area correlate with reduced grip strength and impaired rotarod activity in Ctns^−/−^ mice than in WT and CKD mice (*Figure* 2D and 2E). Reduced cross‐sectional area of muscle is not always associated with diminished muscle strength in cachectic patients.^18^, ^19^

Our results suggest that loss of lean mass, decreased muscle fibre cross‐sectional area, and impaired muscle function are more severe in 12‐month‐old Ctns^−/−^ mice than in age‐matched pair‐fed CKD controls. Indeed, muscle wasting in children with nephropathic cystinosis seems to be more prevalent than in CKD children with comparable degree of renal dysfunction. Although there is no direct comparison, muscle wasting is highly prevalent in patients with nephropathic cystinosis with predialysis CKD (33–60% in two long‐term follow‐up studies).^4^, ^5^ On the other hand, a recent study showed no appendicular lean mass deficits in children with mild to moderate CKD.^20^

We studied muscle mass regulatory signaling pathways in Ctns^−/−^ mice. mRNA contents of Pax‐3 and Pax‐7, Myogenin, and MyoD were decreased in the gastrocnemius muscle of 12‐month‐old Ctns^−/−^ mice (*Figure* 3A), suggesting altered satellite cell content or activity and reduced myoblast differentiation in Ctns^−/−^ mice. Expression of muscle IGF‐I was down‐regulated while Myostatin mRNA was up‐regulated in Ctns^−/−^ mice (*Figure* 3A and 3B). IGF‐I and Myostatin play stimulatory and inhibitory roles, respectively, in the regulation of muscle mass.^21^ Expression of muscle atrophy‐associated genes, Atrogin‐1 and MuRF‐1, was significantly increased in Ctns^−/−^ mice relative to WT controls (*Figure* 3B). Muscle protein contents of IL‐1α, IL‐6, and TNF‐α were significantly increased in Ctns^−/−^ mice than in WT controls (*Figure* 3C). Inflammatory cytokines have been implicated in the pathogenesis of muscle wasting.^22^, ^23^

Skeletal muscle accounts for 20–30% of overall energy consumption at rest.^24^, ^25^ Chronic inflammation in skeletal muscle may cause hypermetabolism in Ctns^−/−^ mice. We showed that muscle inflammatory cytokines (IL‐1α, IL‐6, and TNF‐α) were significantly increased in Ctns^−/−^ mice (*Figure* 3 *C*). Common causes of chronic inflammation include deterioration of renal function, volume overload, alternation in body composition, and acidosis.^22^, ^23^ Metabolic acidosis is unlikely the culprit in Ctns^−/−^ mice as they were not acidotic. Serum bicarbonate level in Ctns^−/−^ mice was not different from that in controls (*Table* 1).

Recent studies suggest that UCP‐1 contributes to adaptive thermogenesis while UCP‐2 and UCP‐3 are involved in the resting metabolic rate.^26^ We investigated the mechanisms of hypermetabolism in Ctns^−/−^ mice. Protein contents of UCP‐2 and UCP‐3 were increased in muscle and WAT of Ctns^−/−^ mice than in WT controls (*Figure* 5A and 5E). In contrast, ATP contents in muscle and adipose tissue were decreased in Ctns^−/−^ mice relative to controls (*Figure* 5B and 5F). In addition, increased UCP‐1 and decreased ATP contents in adipose tissue were observed in Ctns^−/−^ mice than in WT controls (*Figure* 5C, 5D, 5G, and 5H). Adipose tissue is also important in energy metabolism. We, and others, have previously described the increased thermogenesis and up‐regulation of UCPs in adipose tissue in rodent models of cachexia and in cachectic patients.^9^, ^13^, ^27^, ^28^, ^29^, ^30^, ^31^ Up‐regulation of UCPs expression promotes proton leak and reduces cellular ATP production in exchange for the generation of heat.^32^, ^33^ *In vitro* studies have reported that modest increase in the expression of UCP‐2 leads to rapid fall in mitochondrial membrane potential and a reduction of intracellular ATP content.^34^ UCP‐3 modulates the activity of sacro/endoplasmic reticulum Ca^2+^‐ATPase and decreases mitochondrial ATP production.^35^ Several studies have reported decreased levels of ATP in cystinotic cells, including fibroblast, leucocytes, and renal epithelial cells.^36^ Abnormal ATP production has been associated with impaired respiratory chain complex I activity in cystinotic cells.^37^

We also profiled expression of genes related to energy consumption in skeletal muscles of Ctns^−/−^ mice. PPARα and PPARδ as well as CPT1α and CPT1β are key regulators of fatty acid oxidation in muscle.^38^, ^39^ Transcriptional levels of PPARα, PPARδ, and CPT1α were significantly up‐regulated in the skeletal muscle of Ctns^−/−^ mice (*Figure* 6A). This profile has been associated with increased resting metabolic rate and maintenance of leanness in humans.^40^ Activated fatty acid oxidation is associated with up‐regulation of PGC1α and PGC1β, two key molecules involved in the regulation of mitochondrial energy metabolism.^41^, ^42^ This was also confirmed in Ctns^−/−^ mice (*Figure* 6A). In addition, we demonstrated increased expression of thermogenic genes in BAT, and epididymal and inguinal WAT of Ctns^−/−^ mice (*Figure* 6B, 6C, and 6D). UCP‐2 and UCP‐3 regulate fatty acid metabolism in skeletal muscle and adipose cells. UCP‐2 and UCP‐3 promote fatty acid metabolism by exporting fatty acid anions outside of the mitochondrial matrix.^26^ Collectively, our results suggest that up‐regulation of UCPs, elevated key molecules of fatty acid oxidation, and mitochondrial energy metabolism in muscle and adipose tissue may be implicated in the pathogenesis of hypermetabolism in Ctns^−/−^ mice.

Adipose tissue browning has recently been reported in cancer cachexia.^31^ White fat depots contain pockets of UCP‐1‐expressing multiocular cells, called beige (or brite) cells, that can be stimulated on exposure to cold or other stimuli via the process termed browning.^43^, ^44^, ^45^ Epididymal WAT predominantly contains white adipocytes while inguinal WAT contains a mixed population of white and beige adipocytes.^46^ We demonstrated increased expression of unique beige adipose cell markers (CD137, Tmem26, and Tbx1) and BAT marker UCP1 in inguinal WAT of Ctns^−/−^ mice (*Figures* 5G and 7A). Our results suggest the development of beige adipocytes in Ctns^−/−^ mice. The development of browning and beige adipocytes in Ctns^−/−^ mice is a novel finding. The mechanism is currently unknown. Recent evidence shows that transdifferentiation of white adipocytes and *de novo* differentiation of beige adipocyte coexist.^46^ Beige adipocyte precursors have also been identified in skeletal muscle,^47^ which further underlies the complexity of beige adipogenesis. Transcriptional regulator PPARγ is necessary and sufficient for adipogenesis. The actions of PPARγ are modulated by a large set of proadipogenic transcriptional cofactors.^46^ We demonstrated increased expression of PPARGC1α, CIDEA, PDRM16, and DIO2 in inguinal WAT of Ctns^−/−^ mice (*Figure* 6D). The important roles of transcriptional factors DIO2, PDRM16, PPARGC1α, and CIDEA on adipogenesis and thermogenesis have been described.^46^, ^48^ Overexpression of these transcriptional cofactors in WAT favors adipocyte browning and thermogenesis.

WAT browning is responsible for a significant increase in total energy expenditure.^49^ Several mechanisms have been proposed for WAT browning, including activation of Cox2 signaling pathway and chronic inflammation. Activation of Cox2, a downstream effector of β‐adrenergic signaling, is crucial for the induction of brown fat‐like cells in WAT depots.^50^ Cox2 produces prostaglandins that enhance mitochondrial biogenesis and increase the uncoupling capacity when activated with adrenergics.^51^ We showed that inguinal WAT gene expression of Cox2 and PGF2α was significantly increased in Ctns^−/−^ mice vs. WT control mice (*Figure* 7B). Chronic inflammation is a hallmark of both clinical and experimental cachexia.^22^, ^23^, ^52^, ^53^ Inflammatory cytokines, such as IL‐6, play an important role in the WAT browning phenotype in mouse models of cachexia.^54^ Gene expression of IL‐1α, IL‐6, and TNF‐α was significantly elevated in inguinal WAT of 12‐month‐old Ctns^−/−^ mice than that in age‐matched WT controls or CKD mice (*Figure* 7B). Inflammation in WAT is characterized by recruitment of macrophages, including activated M1 and M2 macrophages.^55^ Activated macrophages in the WAT are an important source of adrenaline and noradrenaline and have been associated with an increase in uncoupled respiration and energy expenditure in mice.^56^ We showed that the phenomenon of adipose tissue browning was more advanced in 12‐month‐old Ctns^−/−^ vs. CKD controls, as indicated by the higher expression of browning markers CD137 and Tbx1 (*Figure* 7A). At the same time, there was evidence of more severe inflammation in the Ctns^−/−^ mice compared with CKD controls as indicated by the inflammatory cytokine expression levels of IL‐1α, IL‐6, and TNF‐α in inguinal WAT (*Figure* 7B). We postulate that inflammation is an important mechanism underlying the adipose tissue browning in INC.

## Conclusion

We describe muscle wasting and adipose tissue browning in a mouse model of INC. We show that Ctns^−/−^ mice exhibit disturbances in energy homeostasis before the onset of their CKD. We demonstrate aberrant muscle mass regulatory signaling pathways in Ctns^−/−^ mice. We show that hypermetabolism in Ctns^−/−^ mice is associated with up‐regulation of key enzymes regulating thermogenesis in skeletal muscle and adipose tissues. Importantly, we report novel findings in the development of beige adipocytes in Ctns^−/−^ mice. Further studies are required to investigate the underlying mechanisms of these metabolic defects in INC, which are associated with poor quality of life and mortality, and for which there is no current therapy.

## Acknowledgements

The authors certify that they comply with the ethical guidelines for authorship and publishing of the *Journal of Cachexia, Sarcopenia and Muscle* (*von Haehling S*, *Morley JE*, *Coats AJS*, *Anker SD*. *Ethical guidelines for authorship and publishing in the Journal of Cachexia*, *Sarcopenia and Muscle*. *J Cachexia Sarcopenia Muscle*. *2010*;*1*:*7–8*.)

We gratefully acknowledge Professor Corinne Antignac for providing the Ctns^−/−^ mice. This work is supported in part by funding from Cystinosis Research Foundation. R.H.M. received funding from National Institutes of Health (NIH) U01DK03012; S.C. received funding from NIH Grant RO1‐DK090058 and RO1‐DK099338. P.Z. was supported by Education Department of Heilongjiang province (no. 12541324), Harbin Science & Technology Bureau (no. 2014RFXYJ077), and China Scholarship Council (no. 201308230141). R.L.L. received funding from NIH Grant R24‐HD050837. This work was supported by NIH Grant R24‐HD050837 to create the National Skeletal Muscle Research Center (NSMRC) at University of California, San Diego.

## Conflict of interest

The authors declare they have no conflicts of interest.
